# Free metatarsophalangeal joint to metacarpophalangeal joint transfer – a unique surgical technique

**DOI:** 10.1093/jscr/rjae646

**Published:** 2025-01-31

**Authors:** Zoha Asghar, Zubaid Moazzam Sheikh, Muhammad Asif

**Affiliations:** Department of Surgery, Aga Khan University, National Stadium Road, 74800 Karachi, Pakistan; Department of Medicine and Surgery, Ziauddin University, Shahrah-e-Ghalib Road, 75600 Karachi, Pakistan; Department of Plastic Surgery, Cancer Foundation Hospital, Gulshan-e-Iqbal, 75300 Karachi, Pakistan

**Keywords:** hand and micro reconstructive surgery, plastic surgery, giant cell tumor, metacarpal bone

## Abstract

Giant cell tumors of metacarpal bones is a rare occurrence. The tumor particularly exhibits an aggressive behavior in small bones of the extremities. Different techniques such as curettage with or without bone grafting, en bloc resection (more commonly preferred) and reconstruction have been discussed but a unique way of reconstruction is using the metatarsal for substitution. Other options include the fibular graft or using a portion of the iliac crest. We report the case of a young female with a giant cell tumor of the fourth metacarpal bone where we reconstructed the diseased joint with the metatarsal bone while achieving good local control, preserving lower limb and hand function along with cosmesis. The transfer of osteoarticular ligamentous complexes of the metatarsal bone for reconstruction of metacarpal bone defects is a technique that provides good cosmetic outcomes, excellent function with minimum effects to the donor site function.

## Introduction

Giant cell tumors (GCTs) of the bone account for ~5% of the primary bone tumors [1]. They often affect young adults between the ages of 16 and 35 years and are more common in females. About 85%–90% are in long bones, with 50%–60% in the knee area [2]. Small bones of the hand and foot are rarely affected but when involved the behavior is more aggressive. It typically appears as an expansile, eccentrically placed lytic area because of intratumoral hemorrhage. The lesion normally involves the epiphysis, adjacent metaphysis, and there is frequent extension to the subchondral plate, sometimes involving the joint [3]. We report a unique case of successful transplantation of a second metatarsophalangeal joint (MTPJ) to reconstruct the fourth metacarpophalangeal joint (MCPJ) which was excised due to GCT of the fourth MCP. This technique allowed us to maintain the functionality of the MTPJ of the hand while providing excellent local control.

## Case report

**Figure 1:**
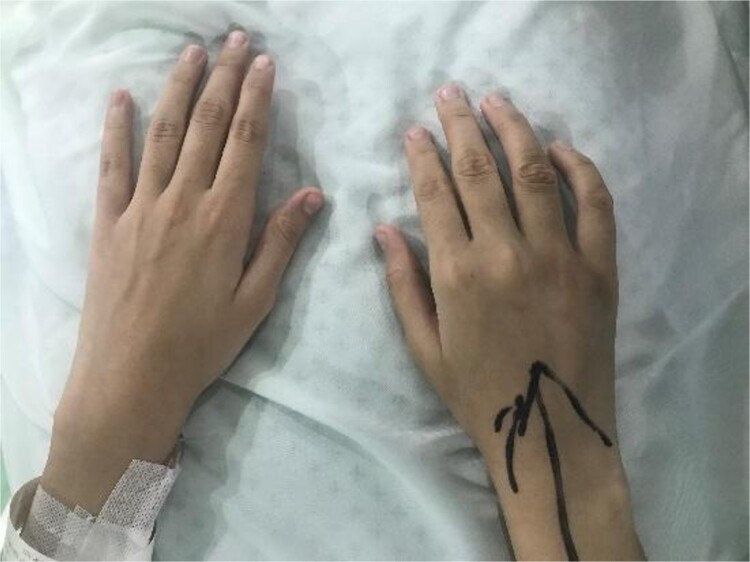
Preoperative photograph of hands showing swelling in fourth metacarpal of the right hand.

**Figure 2:**
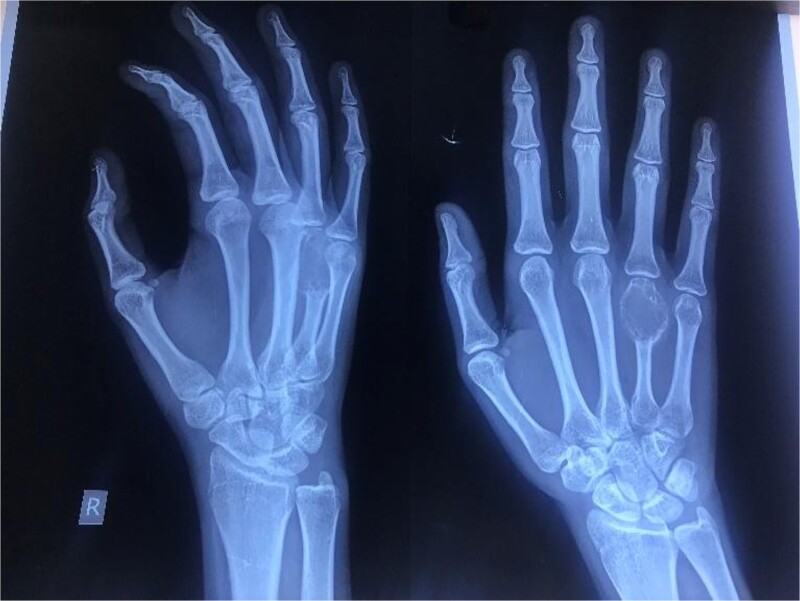
Oblique (left) and antero-posterior (right) view of the right hand revealing an expansile, lytic lesion with a soap bubble appearance over the entire fourth metacarpal bone with thinning of the cortex.

A 20-year-old female, with an unremarkable past medical and surgical history, presented to our clinic with a progressively increasing, painful swelling on the right-hand dorsum on the fourth distal MCP region (Fig. 1). Local examination revealed a 3×3 cm solid immobile swelling at the distal fourth MCP which was painful upon complete extension and flexion. A plain radiograph (Fig. 2 arrow) showed the hallmark expansile soap bubble lesion involving the entire distal fourth MCPJ approaching the articular surface of the MCP. The patient’s routine lab workup was unremarkable. She was counseled regarding all options to make an informed decision and opted for a wide local excision of the lesion along with reconstruction using a free MTPJ transfer.

**Figure 3:**
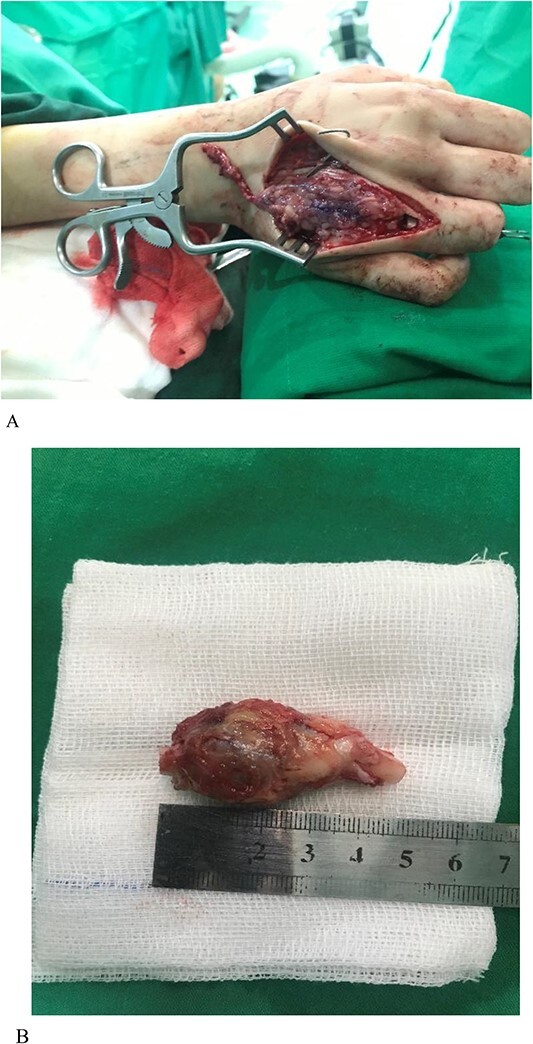
(A) GCT of the metacarpal. Note that the osteotomy of the proximal phalanx is visible in the picture. (B) Lesion excised. Both ends of the bone can be seen.

The patient underwent en bloc resection of the tumor consisting of the fourth metacarpal, MCPJ and a portion of proximal phalanx by dorsal approach (Fig. 3A and B). An osteotomy of MCP was done at the level of the body ~3 cm proximal to the lesion (Fig. 3A).

**Figure 4:**
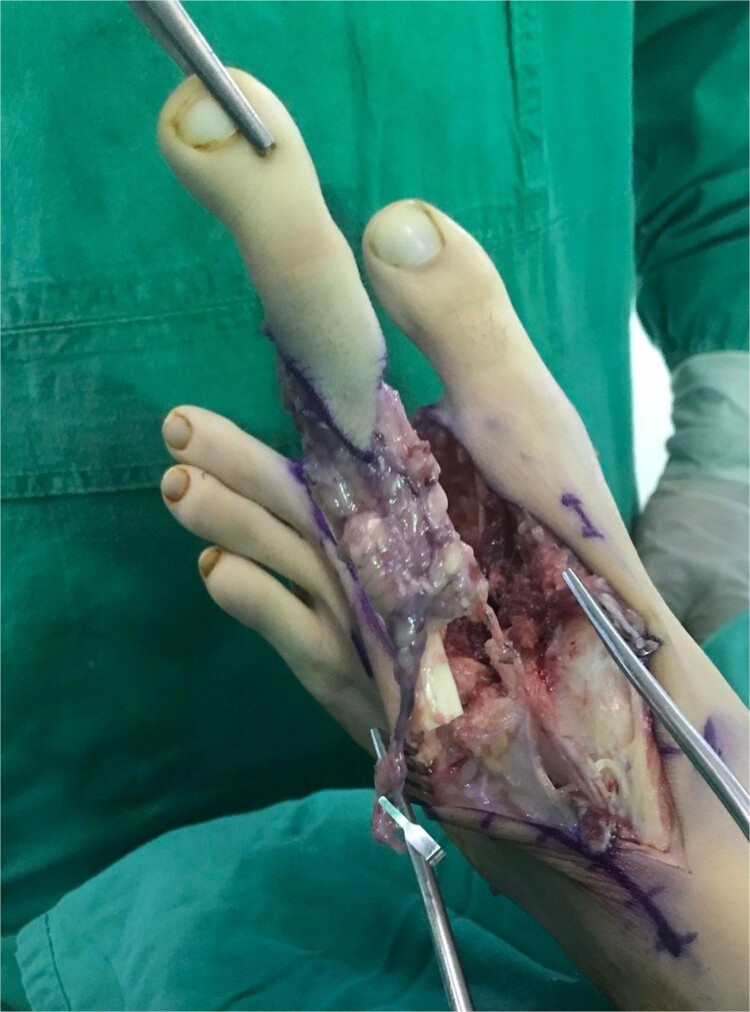
Harvested second toe can be seen.

While excising the affected bone, the distal half of the MCPJ capsule, collateral ligaments, and the whole volar plate were preserved. Subsequently, disarticulation was done at the carpometacarpal joint level. For reconstruction of the resultant defect the left second metatarsal was utilized. A standard second toe harvest along with ~4 cm of the metatarsal bone was performed (Fig. 4). After the harvest was complete the required length of the proximal phalanx was taken from the flap.

**Figures 5:**
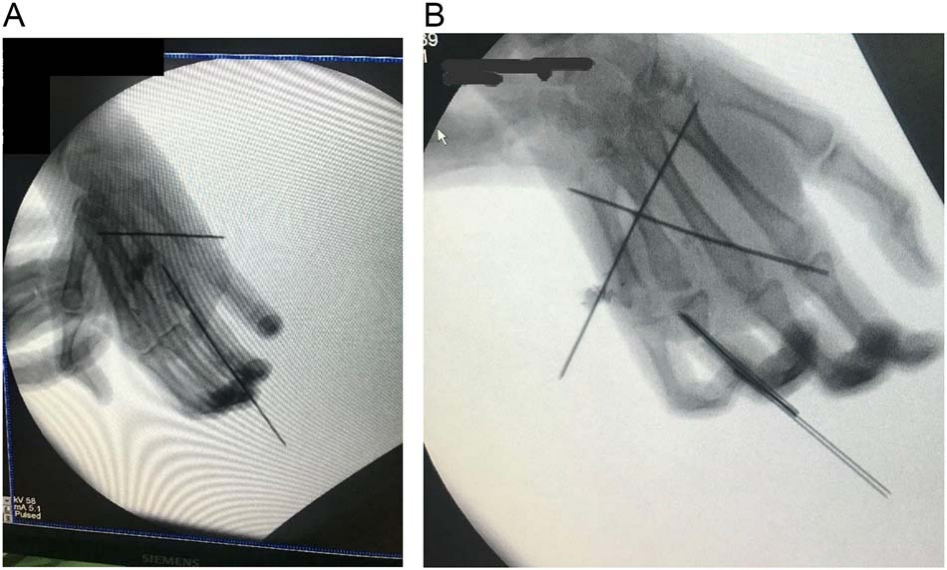
(A) Intraoperative X-ray showing K-wire inserted through the interphalangeal joint crossing the bone flap reaching up to the second metacarpal and another K wire inserted through the interphalangeal joints. (B) After fixation.

The metatarsal was fixed with the base of the metacarpal using Kirschner wire (K-wire) (Fig. 5A and B). An anastomosis was formed between the ulnar artery and the basilic vein. The artery was anastomosed with the common digital artery of the third web-space and two veins were anastomosed. A 1 deep vein was identified, and the other vein was anastomosed in the superficial venous system on the dorsum of the hand (Fig. 5A and B).

**Figure 6:**
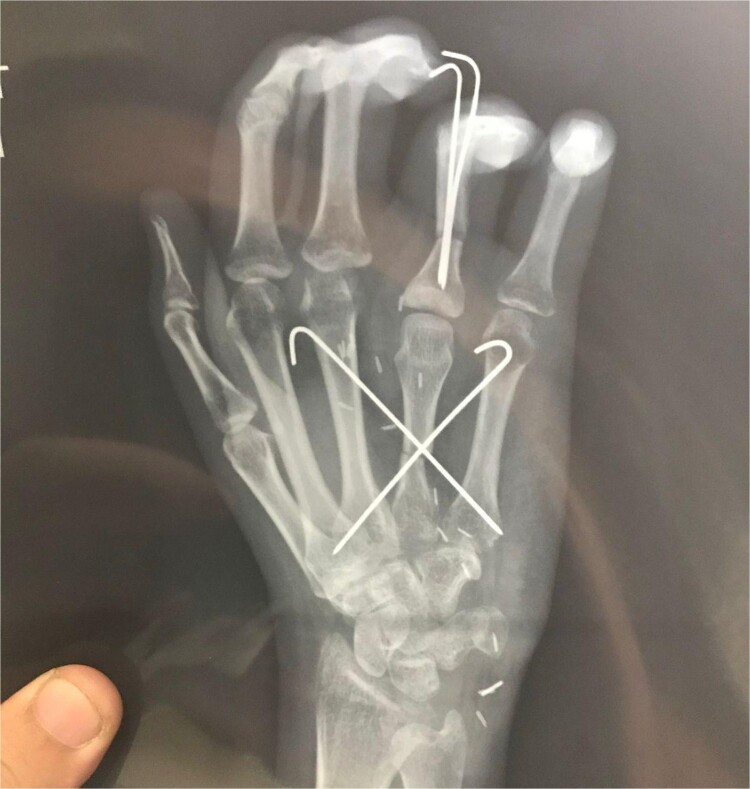
Post-operative X-ray at 3 weeks.

**Figures 7:**
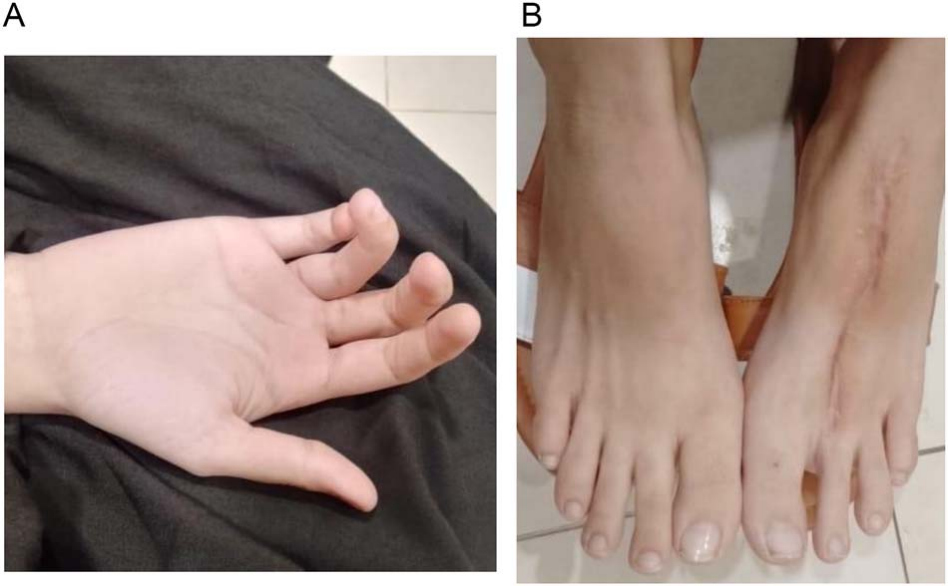
(A) Picture taken at 8 weeks postoperatively of the hand. (B) Picture taken at 8 weeks postoperatively of the donor site.

Post operatively the patient remained stable. The hand was kept in a dorsal back slab with bulky dressing with a hole cut out on the dressing for flap monitoring via handheld Doppler. Flap was monitored regularly over the arterial and venous anastomosis, already marked per operatively and doppler signals remained normal. Patient was discharged on the 4th postoperative day. Donor site wound showed good healing progress and no complications were noted. Postoperative X-rays showed satisfactory healing at both bony unions (Fig. 6). Regular dressings were done, K-wires were removed at 6 weeks and the patient was referred for physiotherapy of the small joints of the hand to regain functions. The patient showed good movement at the MCPJ at the follow up visits. She was able to do house chores and write normally. On examination there was slight medial rotation of the ring finger which the patient compensated by using her thumb. There was also slight flexion deformity of the distal interphalangeal joint of the ring finger (Fig. 7). No evidence of local or distant metastasis was present.

## Discussion

To the best of our knowledge, this is the first case to be reported across Pakistan. There are only a few reports available regarding the treatment of these aggressive lesions by en bloc excision and osseous replacement. Sporadic case reports have advocated the use of iliac bone or fibula [4, 5]. Pardo-Montaner *et al.* described two cases of successful transplantation of a metatarsal to a metacarpal, which was the site of a recurrent giant-cell tumor. The patients had satisfactory results 3 years later without problems in the foot. They recommended that en bloc resection of the tumor and reconstruction with an autograft should be considered in the treatment of recurrent GCT of the hand [6]. Kotwal *et al.* [7] reported two cases of recurrent GCT of the head of the second metacarpal treated by marginal excision and reconstruction with vascularized toe joint transfers. The stresses across the small joints are much less than that of the weight-bearing joints of the lower extremities. This should favorably affect the outcome of transplanted cartilage in the upper extremity. The template effect of the opposing normal articular surface and the remaining synovial tissue in this hemi-joint transfer also contribute to good outcomes. Denervation of the joint may accelerate degenerative changes, but this effect is diminished in hemi-joint transfers providing better outcomes. Literature supports that despite cartilage destruction, the functional recovery obtained is sufficient to consider joint transfers to salvage the prehensile function of the hand [8]. Another case series from India concluded that the osteoarticular replacement with metatarsal grafting after en bloc resection to treat expansile osteolytic lesions of metacarpals provided very good and consistent functional outcomes without any recurrence [9]. These findings are consistent with our results, and we encourage our colleagues to incorporate this technique within their practice of hand and reconstructive surgery.

## Conclusion

The patient in our case was able to achieve favorable results and functionality in the operated hand which supports that en bloc resection with autologous bone transfer is associated with better outcomes as compared to curettage and bone grafting which are associated with higher rates of recurrence. It is a straightforward and safe surgery which we recommend for locally aggressive lesions.

## References

[1] Tarun PK , Harshavardan JK, Vijayaraghavan V. Giant cell tumour of mertacarpal an illustrative case report. IOSR J Dent Med Sci 2016;15:1–7.

[2] Prashant K , Bhattacharyya TD, Frank H, et al. An unusual case of giant cell tumor of first metatarsal: a rare case report and review of literature. J Orthop Case Rep 2016;6:3–6. 10.13107/jocr.2250-0685.604 PMC5404157 28507956

[3] Saikia KC , Bhattacharyya TD, Bhuyan SK, et al. Local recurrences after curettage and cementing in long bone giant cell tumor. Indian J Orthop 2011;45:168–73. 10.4103/0019-5413.77138. 21430873 PMC3051125

[4] Carlow SB , Khuri SM. Metacarpal resection with a contoured iliac bone graft and silicone rubber implant for metacarpal giant cell tumor: a case report. J Hand Surg 1985;10:275–8. 10.1016/S0363-5023(85)80121-0. 3980944

[5] Athanasian EA , McCormack RR. Recurrent aneurysmal bone cyst of the proximal phalanx treated with cryosurgery: a case report. J Hand Surg 1999;24:405–12. 10.1053/jhsu.1999.0405. 10194029

[6] Pardo-Montaner J , Pina-Medina A, Barcelo-Alcaniz M. Recurrent metacarpal giant cell tumour treated by en bloc resection and metatarsal transfer. J Hand Surg 1998;23:275–8. 10.1016/s0266-7681(98)80197-6 9607682

[7] Hutter RVP , Worcester JN Jr, Francis KC, et al. Benign and malignant giant cell tumors of bone. A clinicopathological analysis of the natural history of the disease. Cancer 1962;15:653–90. 10.1002/1097-0142(196207/08)15:4<653::AID-CNCR2820150402>3.0.CO. 14450279

[8] Menon J . Reconstruction of the metacarpophalangeal joint with autogenous metatarsal. J Hand Surg 1983;8:443–6. 10.1016/S0363-5023(83)80206-8. 6886340

[9] Pallapati SCR , Thomas BP, Anderson GA. En bloc excision and matched metatarsal transfer for expansive benign osteolytic lesions of the metacarpal. J Hand Surg 2016;41:e417–23. 10.1016/j.jhsa.2016.08.004. 27614921

